# Numerical Simulations of the Soil–Rock Mixture Mechanical Properties Considering the Influence of Rock Block Proportions by PFC2D

**DOI:** 10.3390/ma14185442

**Published:** 2021-09-20

**Authors:** Wenwei Gao, Hairong Yang, Le Wang, Ruilin Hu

**Affiliations:** 1 Department of Architecture and Engineering, Yan’an University, Yan’an 716000, China; 2 Institute of Language Literature and Mass Media, Yan’an University, Yan’an 716000, China; yadx_yhr@yau.edu.cn; 3 Institute of Hydrogeological and Engineering Geological Survey, Gansu Geology and Minerals, Zhangye 734000, China; wangle@gsdzgckcy999.onexmail.com; 4 Key Laboratory of Shale Gas and Geo-Engineering, Institute of Geology and Geophysics, Chinese Academy of Sciences, Beijing 100029, China; hurl@mail.iggcas.ac.cn; 5 College of Earth and Planetary Sciences, University of Chinese Academy of Sciences, Beijing 100049, China

**Keywords:** soil-rock mixture (S-RM), particle flow code (PFC), numerical simulation, mechanical characteristics, rock block rotation

## Abstract

Soil-rock mixtures (S-RMs), as a kind of special engineering geological material, need to be studied because of the special structure and complex movement mechanism of their rock blocks, their physical and mechanical properties, and the factors underlying rock block movement in the process of their deformation and failure. In this paper, a series of discrete-element numerical models are constructed in particle flow code software (PFC^2D^). First, the random structure numerical models of S-RMs with different rock block proportions are established. Then, the parameters of the soil meso-structure are inversed by the biaxial simulation test, and a series of biaxial compressive tests are performed. The characteristics of stress and strain, deformation and failure, and rock block rotation and energy evolution are systematically investigated. The results show the following. (1) As the rock block proportion (confining pressure 0.5 MPa) increases, the peak strength of increases, the fluctuations of the post-peak become more obvious, and the dilatancy of the sample increases. (2) As the rock block proportion increases, the width of the shear band increases, the distribution of cracks becomes more complex and dispersed, and the range of the shear zone increases. (3) The number of rock blocks with rotation also increases significantly as rock block proportion increases, and the rotation angles are mostly between −5° and 5°. (4) The strain energy of S-RMs with different rock block proportions follows the same change rule as axial strain, showing a trend of first increasing and then decreasing, like the stress–strain curve.

## 1. Introduction

As a special geological material, soil-rock mixtures (S-RMs) refer to inhomogeneous mixed media embedded in high-strength rock blocks in a relatively low-strength, fine-grained matrix [1,2,3,4,5]. S-RMs are geological bodies characterized by a low degree of consolidation, low strength, a complex soil–rock structure, and high water sensitivity. Consequently, S-RMs as a type of medium are susceptible to widespread landslides and are the principal carrier of slope disasters in mountainous areas. Hence, studying the mechanical properties of S-RMs has important engineering value and theoretical significance [6,7].

S-RMs are composed of soil and rock blocks that differ in their distribution patterns and strength. As a result, S-RMs are nonhomogeneous, discontinuous, and anisotropic. In addition, with regard to physical and mechanical properties, S-RMs are extremely nonlinear, as well as highly environmentally sensitive and dependent [8,9,10,11]. The deformation and failure patterns as well as mechanical properties of S-RMs depend on their mesoscale characteristics, such as the physical and mechanical properties of the internal fine-particle-phase soil; the proportion, spatial distribution, and morphology of the internal rock blocks; and the properties of the contact surfaces between the rock blocks [12,13,14]. The investigation of the deformation and failure characteristics of S-RMs from a mesoscale perspective, and the analysis of S-RMs through mesomechanical methods with the goal of determining their deformation and failure modes under loading have become trends in research [15,16].

With the advancement of computer technology, many numerical simulation methods have investigated the deformation and failure mechanism of S-RMs [17,18]. Among them, the finite element method (FEM) [10,19,20] and the finite difference method (FDM) [21,22,23] are mainly used to study the deformation and mechanical properties of S-RM. Unfortunately, it is difficult to employ FEM and FDM to accurately depict the deformation and failure characteristics of S-RMs, which are nonhomogeneous, discontinuous materials. In addition, these two methods cannot be used to determine the mesoscale effects of factors related to rock blocks (e.g., their morphology and proportion) on the progressive failure process. In recent years, the discrete element method (DEM) has been increasingly applied to the deformation and failure analysis of S-RMs. In particular, the particle flow code (PFC), a method developed based on discrete element theory, has shown application potential [24,25]. From the perspective of the mesomechanical properties of loose media, the PFC method analyses the macroscopic physical and mechanical behaviour of materials by simulating of the motion and interaction of granular media. This method has considerable advantages for solving problems related to interparticle interactions and large deformations [26,27,28].

In this study, the numerical simulation software particle flow code (PFC^2D^) was used to analyse the deformation and failure characteristics of S-RMs. First, the numerical models of S-RMs with different rock block proportions were established. Then, the parameters of the soil meso-structure were inversed by the biaxial simulation test. Finally, using biaxial tests in PFC^2D^, a series of numerical analyses were performed to investigate the mechanical behaviours and failure mechanism of the S-RMs with different rock block proportions.

## 2. Setup of Numerical Models

### 2.1. Generation of the Irregular Rock Blocks

PFC^2D^ provides a clump command for simulating irregular particles. A clump consists of an aggregation of basically spherical particles with fixed relative positions. As the calculation of the contact between the particles composing a clump is skipped during cycling, the computational time is considerably reduced [29]. The particles at the edge of a clump interact with the particles outside of it. However, a clump does not disintegrate regardless of whether a contact force acts on it. The rock blocks in an S-RM are much stronger and stiffer than a fine-grained soil matrix. Therefore, it is suitable to simulate irregular rock blocks with clumps.

**Figure 1:**
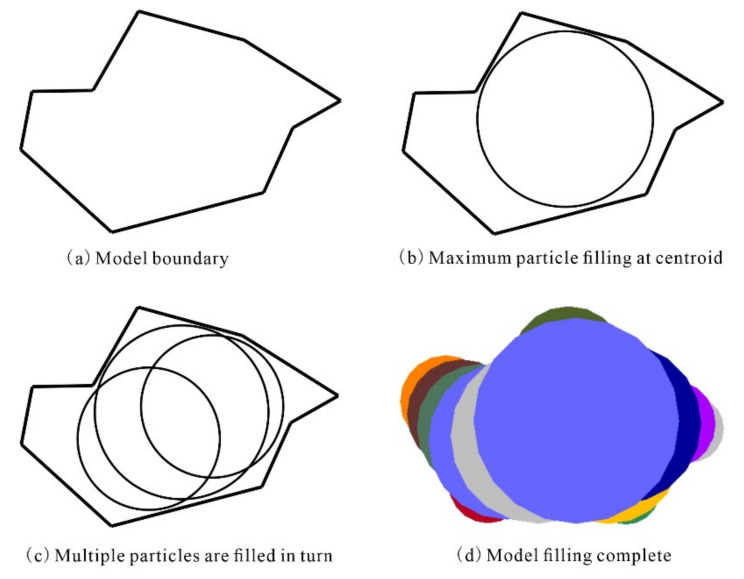
The filling process of the clump model.

The clump command establishes a complex contour through the superimposition of multiple balls, which are often referred to as pebbles to differentiate them from regular particles. To build a complex clump model, a number of methods can be used to fill the clump with pebbles. Overall, these methods are similar in terms of the principle and the basic procedure, which mainly involves the following steps [30]:

- A complex model is often simplified to a polygonal model with a finite number of sides (Figure 1a). An established clump model with many sides needs to be filled with many pebbles and is therefore complex.
- The centroid of the model is first located and then filled with the first pebble to form its core area. This pebble is the largest circle, with the centroid of the model as its centre, and does not contain any of the boundary points.
- For each boundary point, the angle bisector passing through it is calculated with both its sides as vectors. The interior of the model is iteratively filled with new pebbles at different locations along the angle bisector until a circle in contact with just one of the boundary points is found. This circle is the largest pebble that can be obtained through the contour points (Figure 1b).
- All the outer contour points of the model are traversed through continuous cycling. Of all the possible pebbles, there exists one pebble that allows the largest increase in the internal area of the model. This pebble is then used to fill the model (Figure 1c). This process is then repeated. Of the remaining pebbles, those that allow the largest increase in the internal area of the model are determined one by one and used to fill the model until the required particle coverage is reached, at which point a final model is generated, as shown in Figure 1d.

### 2.2. Principle of Placing Rock Blocks into the Space Domain

At present, there are many methods to randomly simulate the meso characteristics for S-RM [30]. The method adopted in this paper is to set the shape (polygon or circle), roughness coefficient, material type, grading interval, proportion, and generation area of the block stone in the accumulation medium, and then generate the rock blocks according to the block stone particle size from large to small in the region, to ensure the random shape and distribution position of the block and no contact between rock blocks [26].

**Figure 2:**
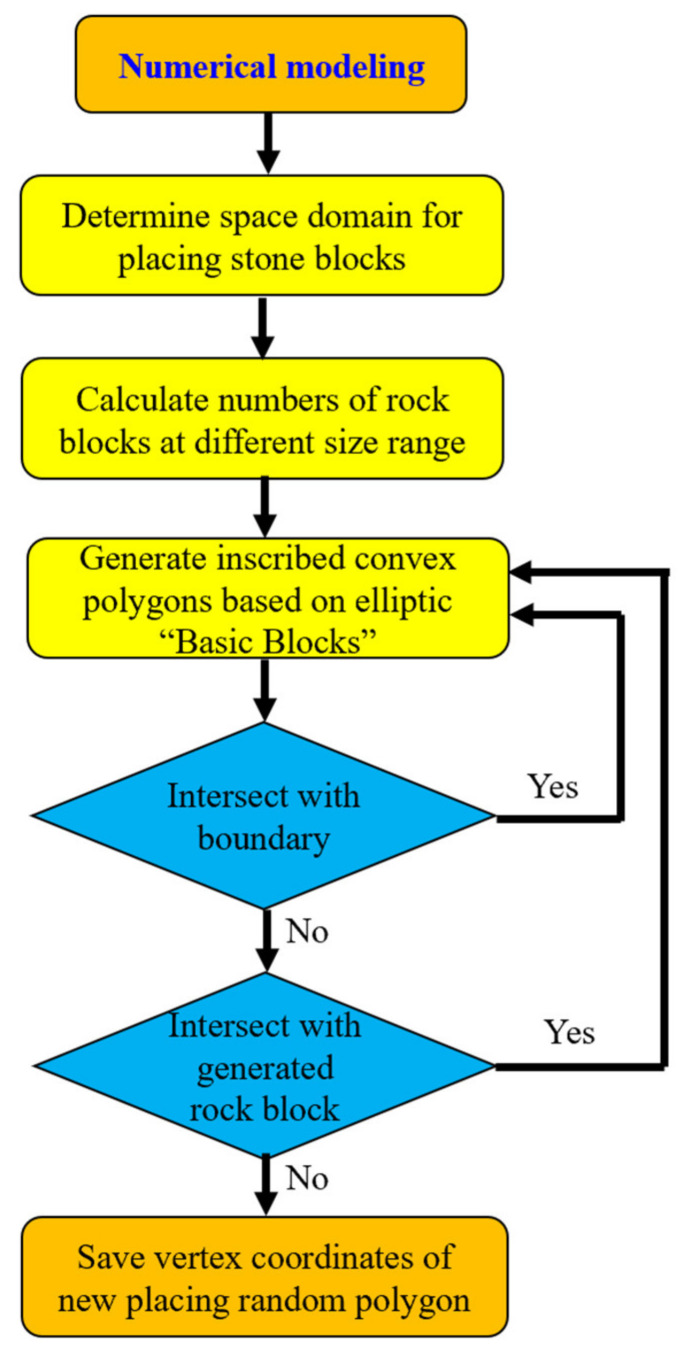
Modeling process of S-RM with random structure.

Figure 2 depicts the procedure of S-RM with random structure. As can be seen, Block placement is a process of trial placement, judging the intersection, adjusting the position, and determining the placement. The placement order of the blocks is based on the gradation particle size from large to small, until the rock-block proportions is met.

### 2.3. Model Dimensions

**Figure 3:**
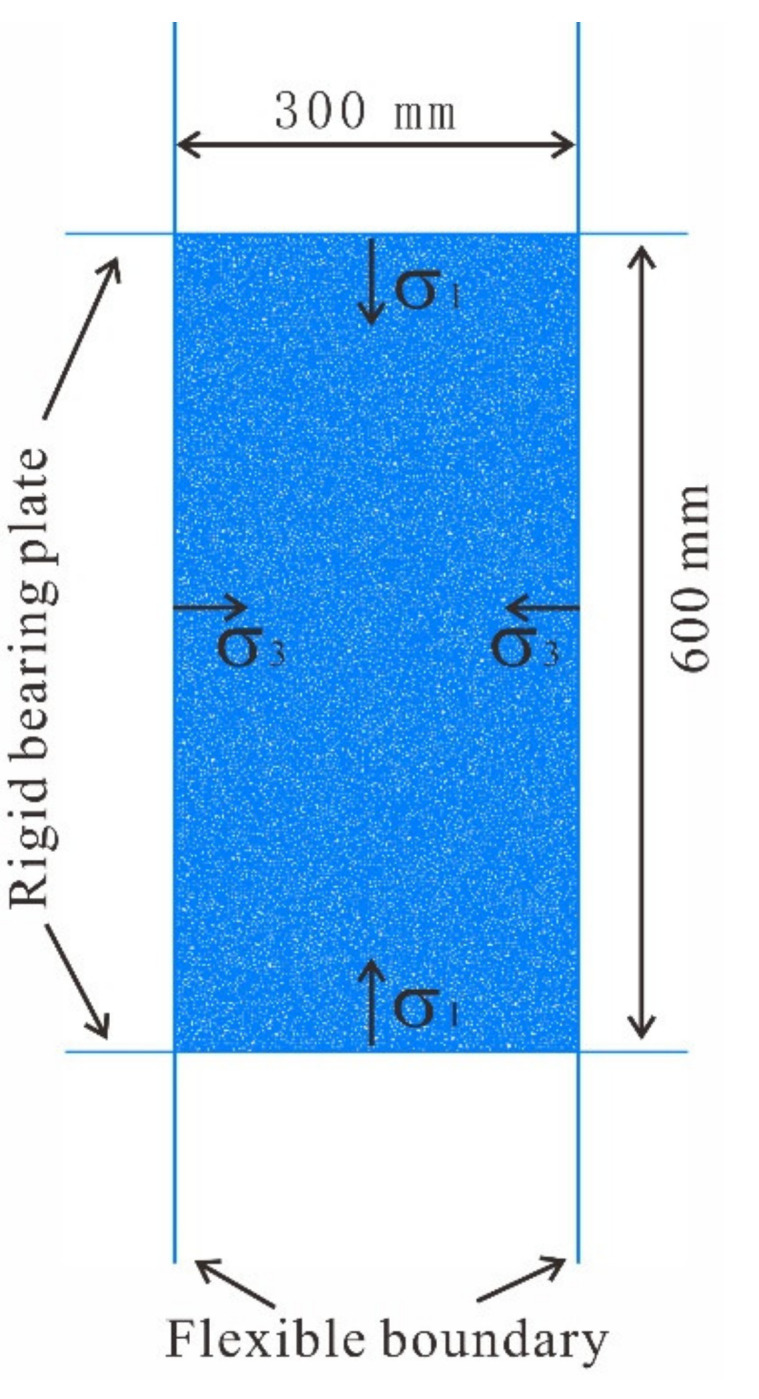
Schematic diagram of the biaxial test.

To date, no unified dimension standards have been established for biaxial test models. In most cases, simulation model dimensions are set based on the specimen dimensions used in relevant laboratory tests and the conclusions drawn from numerical tests. In fact, the specimen dimensions used in laboratory tests are generally used as the simulation model dimensions. Figure 3 shows a geometric diagram of the S-RM specimen model used in the biaxial test conducted in this study. The height–diameter ratio of the specimen was set to 2. Specifically, the diameter and height of the specimen were 300 and 600 mm, respectively. To ensure maximum consistency with the loading process in laboratory testing, a confining pressure and an axial pressure were applied through the walls on the four sides during the test. The rigid loading plates of a triaxial testing machine were simulated with the top and bottom walls, and the contact stiffness was set to 10 times the stiffness of the S-RM. A confining pressure was applied through the left and right walls. During the loading stage of a triaxial test, the confining pressure is generally a flexible constraint. Therefore, the stiffness of the loading plates on both sides was set to 1/10 of that of the soil material. The displacement mode was used to control axial loading. To ensure consistency with the actual loading process, the loading rate was set to 0.02 m/s, and the loading process was terminated at an axial strain of 10%. The particle radius was set to 1.5–2.0 mm. Approximately 30,000 particles were generated.

**Figure 4:**
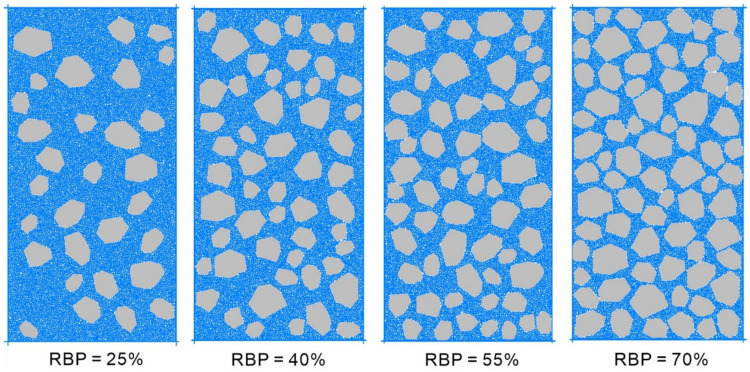
Numerical specimens of the S-RMs.

The concepts of “soil” and “rock” in an S-RM are relative, and Medley and co-workers pointed out that the characteristic size $L_{c}$ of the research object is the basis for determining the size range of “rock blocks” [1], and the soil/rock threshold can be defined as $d_{thr}=0.05\;L_{c}$, where $d_{thr}$ is the soil/rock threshold of the S–RM and $L_{c}$ comprises the dimension of a laboratory specimen [19] ($L_{c}$ was set to 300 mm in this study). Therefore, the minimum size of the rock blocks in the model was 15 mm. Considering the generation efficiency and computational accuracy for the rock blocks, their sizes were simplified to a range of 30–80 mm. In addition, the number of sides of a rock block and the density of the rock blocks, $\rho_{R}$, were set to 4–8 and 2700 kg/m^3^, respectively. A contact-bond model with a coefficient of friction u of 0.5 and normal and tangential strengths of 1.5 kN was used to simulate the soil–rock interfaces. Similarly, a contact-bond model was used to simulate the soil particles. The soil particle size was set to 1.5–2.0 mm. Four models with rock block proportions (RBPs) of 25%, 40%, 55%, and 70% were established (Figure 4). The confining pressure was set to 0.5 MPa.

### 2.4. Calibration of Micro-Parameters

**Figure 5:**
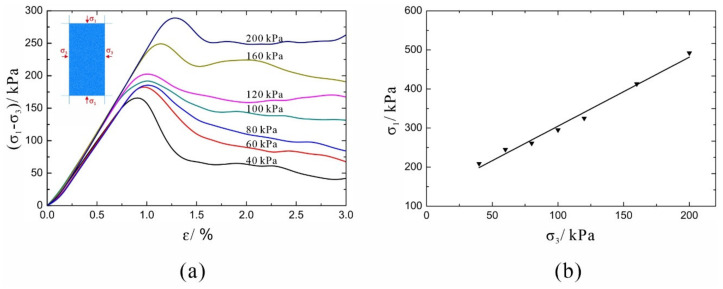
The calibration process of the micro-parameters through biaxial compressive experiments: (**a**) Stress–strain curve of the soil model under different confining pressures. (**b**) Correlation between two principal stresses of the soil model.

The macroscopic parameters of the rock mass are reflected by the microscopic parameters of the interparticle contacts in the PFC. Many numerical experiments need to be carried out to find the best combination of microscopic parameters to match the macroscopic parameters, because of the obvious nonlinear relationship between the macroscopic parameters of the rock mass and the microscopic parameters of the particles. The model trial method is widely used to determine the meso-parameters of particles [31]. The geometric model of the biaxial test is shown in Figure 3. The soil mechanical parameters obtained from the field push-shear test are shown in Table 1. The biaxial compression test with different confining pressures serves as the basis for obtaining the friction angle *φ* and cohesion *c* in soil. After repeated trial calculations, the stress–strain relationship curve of soil under different confining pressures is obtained (Figure 5a), assuming that the failure of soil agrees with the linear Mohr–Coulomb criterion. According to the test, the stress–strain curve of the material is obtained. In addition, the corresponding material strength parameters can be calculated by using Equations (1) and (2). By fitting the strength values under different confining pressures, as shown in Figure 5b, the corresponding relationship is obtained: $\sigma_{1}=1.78\sigma_{3}+128.38$. Moreover, the internal friction angle *φ* = 16.3° and cohesion *c* = 48.1 kPa of the soil can be obtained by performing an inverse calculation. The ultimate micro-parameters of the soil particles are listed in Table 2.

(1) $$\sigma_{1}=N_{\varphi }\sigma_{3}+2c\sqrt{N_{\varphi }}$$

(2) $$N_{\varphi }=\sqrt{\frac{1+sin\varphi }{1-sin\varphi }}=\tan\left(\frac{\varphi }{2}\right)\;\;\;\;\;(0^{{}^{\circ}}<\varphi <90^{{}^{\circ}})$$

## 3. Analysis of the Mesoscale Mechanism of S-RMs through Biaxial Testing

With regard to mechanical properties, S-RMs are notably distinct from ordinary rock–soil bodies, mainly in areas such as stress–strain and shear dilation/contraction properties, as well as their deformation and failure characteristics. Therefore, in this study, the confining pressure was monitored, and specimen data (e.g., the axial stress and strain, volumetric strain, interparticle tensile and shear cracks, and rock-block rotation) were recorded during the loading process.

### 3.1. Analysis of the Mechanical Effects of S-RMs

#### 3.1.1. Stress–Strain Relations

**Figure 6:**
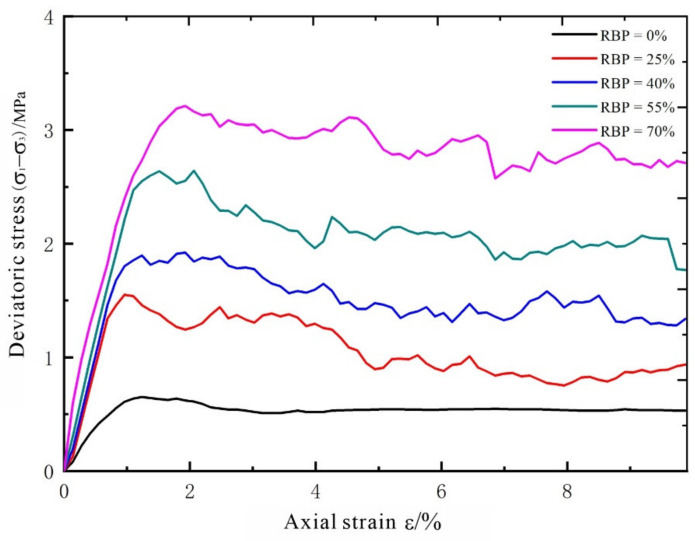
Relationship between stress and strain of S-RMs.

Based on the above simulated biaxial test results, deviatoric stress–axial strain curves were plotted for the S-RM specimens with different RBPs (Figure 6). It can be seen that the stress–strain curve for each S-RM specimen at the initial loading stage is close to a straight line, mainly for the following reason: The particles in the PFC are composed of nondeformable rigid models and are densified during the servo stage. During the initial loading stage, particle embedment is insignificant, so the particles deform in conformity with the bond parameters, which is macroscopically reflected by a nearly straight stress–strain curve. The slope of this straight line is the elastic modulus of the S-RM. As shown by the whole stress–strain curves, under the same confining pressure, the elastic modulus, peak strength, and residual strength of a given S-RM all increase significantly as the RBP increases. During the residual deformation stage, the stress–strain curves for different S-RM specimens exhibit different shapes. An increase in the RBP leads to notable stress jumps, consistent with previous findings. At a low RBP, an increase in the strain induces movement of the soil particles, resulting in the translation and rotation of the rock blocks. However, due to their low proportion, the rock blocks are unable to come in direct contact with each other, and the external force exerted on the specimen is transferred primarily through the soil matrix material. The extent of contact between the rock blocks is relatively small. As the specimen cracks, the rock blocks continuously adjust their relative positions under the axial pressure. However, compared to soil compaction, this nonlinear change is relatively small. As the RBP increases, the rock blocks gradually play a more dominant supporting role. As the strain increases, the rock blocks have a strong tendency to interact with each other. Under these conditions, the soil acts as a filler, and some rock blocks become suspended. The rock blocks play a main role in withstanding the axial load. The majority of the rock blocks are in contact and mesh with each other. As the loading process proceeds, the relative positions of the rock blocks adjust more, which is reflected by the appearance of discontinuous jump points on the stress–strain curve.

According to the analysis of the internal structure of the S-RM, the soil plays a major role in the initial stage of stress, and the blocks only bear part of the load, so the rock blocks in the S-RM are wrapped by the soil, the rock blocks are unable to come in direct contact with each other, which is the typical difference between the S-RM and geological materials.

#### 3.1.2. Volumetric Strain Characteristics

**Figure 7:**
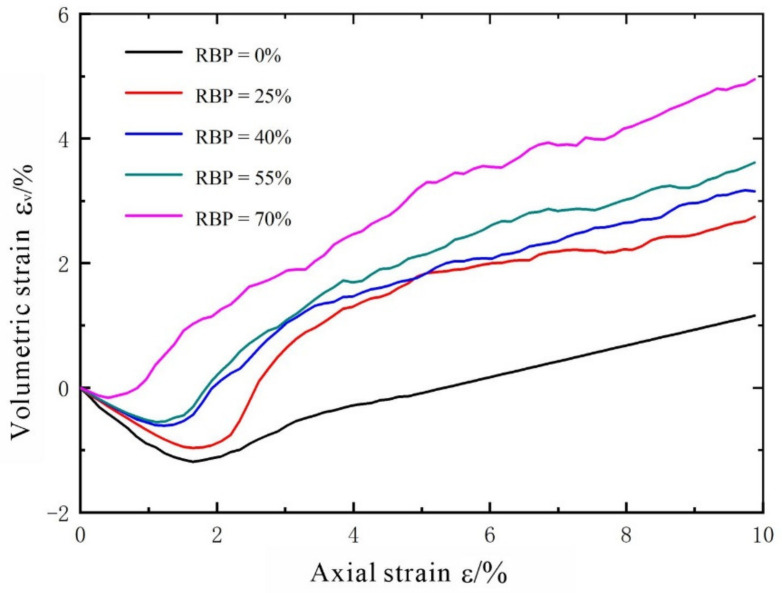
Relationship between volumetric strain and axial strain.

During the biaxial compression process, the volumetric strain of a specimen is generally reflected by a shear dilation and contraction. Figure 7 shows the recorded volumetric strain–axial strain curves for the specimens (a shear dilation and a shear contraction are deemed positive and negative, respectively). A consistent dilation/contraction pattern can be observed in the S-RM specimens with different RBPs. The volume of a specimen first decreases, that is, a specimen first undergoes a shear contraction, the extent of which increases as the RBP increases. As the axial strain increases to 1–2%, the shear contraction gradually stops increasing, followed by a gradual increase in the volumetric strain (i.e., a shear dilation). An increase in the RBP reduces the shear contraction and increases the shear dilation, for the following reason: As the RBP increases, the amount of soil proportionally decreases. The initial shear contraction of a specimen is caused by the compaction of the soil particles. At a high RBP, it takes a short time for the soil to become compacted. As the axial strain increases, relative dislocations occur between the rock blocks, and some rock blocks become suspended, resulting in an increase in the volumetric strain of the specimen. Moreover, a large number of irregular rock blocks leads to a higher shear dilatancy.

### 3.2. Mesoscale Failure Analysis of the Specimens

**Figure 8:**
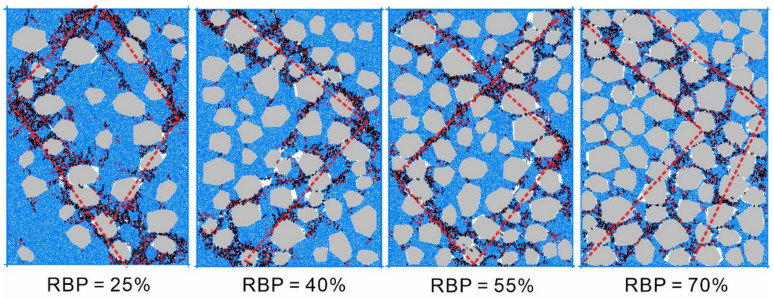
Morphology of shear failure of S-RM.

Figure 8 shows the morphology of the S-RM specimens at failure under a confining pressure of 0.5 MPa and a strain of 10%. The shear-zone distribution patterns show that a high RBP leads to a wide shear zone. At failure, of all the S-RM specimens, the S-RM specimen with an RBP of 70% contains the most widely and complexly distributed cracks and the widest shear zone. In addition, in this specimen, a notable “rock bypassing” phenomenon, as well as collision and contact between the surrounding rock blocks, can be observed in its shear zone, which is distributed in a relatively discontinuous manner. Only the rock blocks near the shear zone have some aggregation and contact, and there is no obvious displacement of the rock blocks outside the shear zone, which is shown in the simulation results. On this basis, the following formation and evolution pattern of cracks in an S-RM arises. First, the soil and rock blocks in an S-RM have significantly different mechanical properties, so their deformation is incompatible, which leads to stress concentration at the soil–rock interfaces. As a result, the soil–rock interfaces become weak links in the S-RM, where first cracks are formed and propagate. Moreover, the presence of the rock blocks prevents crack propagation and coalescence, leading to a complex S-RM failure mode. These two factors constitute the development and evolution mechanism of cracks in an S-RM. The simulation results are similar to the indoor test, but the simulation results can more intuitively show the failure mode [32]. In discrete-element simulations, crack evolution is a visual representation of the deformation and failure of S-RMs, so it can satisfactorily reflect their deformation and failure characteristics.

### 3.3. Analysis of the Rotational Characteristics of Rock Blocks

**Figure 9:**
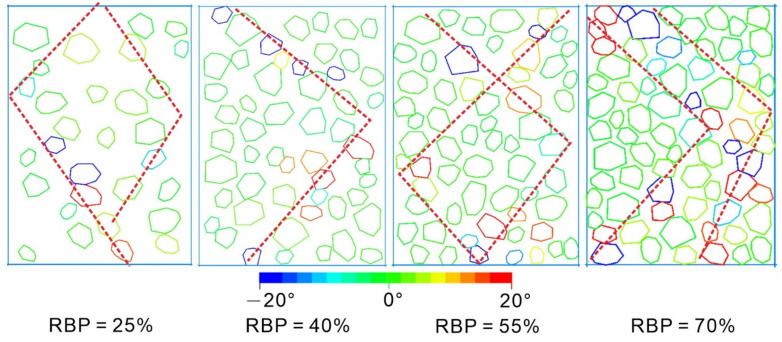
Rock rotations in numerical S-RM specimens.

**Figure 10:**
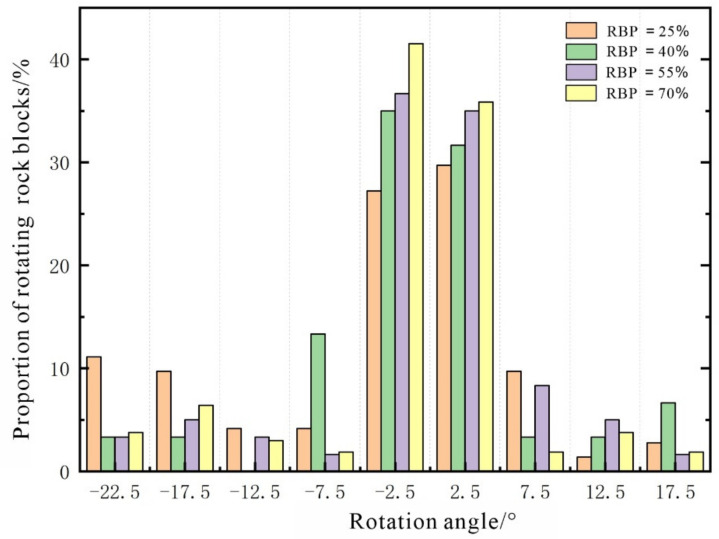
Rock rotation frequency distribution map in numerical S-RM specimens.

The soil and rock blocks in an S-RM undergo both translation and rotation under loading. The rotation of soil particles is an energy dissipation process. An analysis of the rotation of soil particles can determine the energy-dissipation characteristics of the soil. However, energy dissipation from the soil in an S-RM is affected by many factors and does not substantively affect the macroscopic deformation and failure of the mixture. Therefore, the rotation of soil particles is often negligible. In contrast, the rotation of the rock blocks in an S-RM can induce a macroscopic mechanical response from the mixture. During loading, the translation of the rock blocks in an S-RM specimen is generally an integral process. However, the difference in the loading stress often results in a considerable difference in the rotation of the rock blocks between different areas. This localized difference is an important metric of the local deformation characteristics as well as an important reflection of the evolution of the shear zone. Figure 9 shows a schematic diagram of the rotation of the rock blocks in the S-RM specimens (strain = 10%). Figure 10 shows the frequency distribution of the angle of rotation of all the rock blocks at each RBP (strain = 10%) (the anticlockwise and clockwise rotation of rock blocks are deemed positive and negative, respectively).

An analysis of Figure 9 shows the following. At each RBP, rock blocks undergoing notable rotation are concentrated primarily near the shear zone. As the RBP increases, the shear zone expands. All the rock blocks outside the shear zone rotate by very small angles. Therefore, the width of the shear zone can be determined from the rotation of the rock blocks. In addition, the number of rock blocks that undergo shear rotation under loading increases significantly as the RBP increases.

Figure 10 shows the frequency distribution of the angle of rotation of the rock blocks at a strain of 10%. The angle of rotation of the rock blocks ranges mainly from −20° to 20°. Therefore, we set the statistical interval to 5°. Most of the rock blocks rotate by relatively small angles, primarily ranging from −5° to 5°. As the RBP increases, the meshing between the rock blocks strengthens, while their angle of rotation decreases. Consequently, the peak proportion of the rock blocks that rotate gradually increases as the RBP increases. An analysis of the interaction between the rock blocks shows the following. At a low RBP, there is no notable contact between the rock blocks as the specimen undergoes deformation under loading, so the rock blocks can withstand the external force through movement. At a high RBP, the rock blocks are more likely to rotate after collision and meshing with each other. As a result, the rock blocks display notable rotational characteristics at a high RBP.

### 3.4. Analysis of Energy Evolution

**Figure 11:**
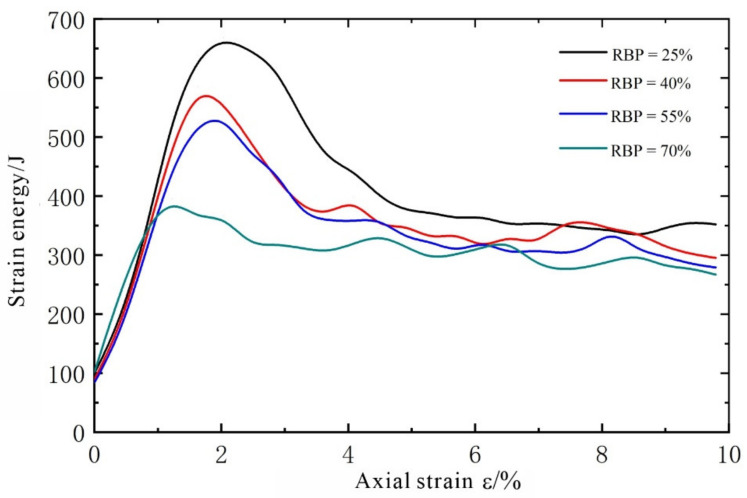
Relationship between strain energy and strain.

**Figure 12:**
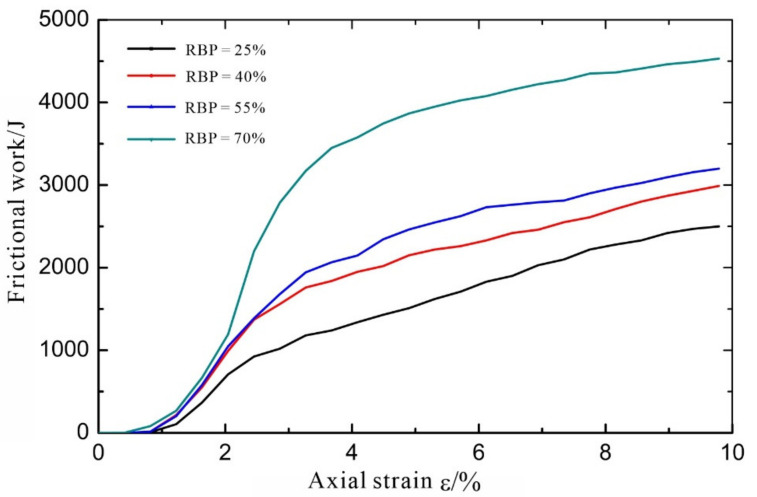
Relationship between frictional work and strain.

In discrete-element simulations, any relative movement between particles can generate a force, and the force between particles can induce a change in energy. The deformation of, and energy dissipation from, the soil in an S-RM reflects the process in which the S-RM resists the external force and reaches a new equilibrium state. The strain energy can be used to reflect the deformation of the soil, while the frictional work done by the rock blocks can be used to reflect the collision, meshing, and sliding between them. The relationships between the strain energy and the frictional work between the rock-block particles, as well as between the strain energy and axial strain, during the test process were determined, as shown in Figure 11 and Figure 12, respectively.

Similar strain energy–axial strain relationships can be observed in the S-RM specimens with different RBPs from Figure 11: The strain energy first increases and then decreases as the axial strain increases, which is similar to the stress–strain relation. In addition, the strain energy decreases as the RBP increases. When RBP = 25%, the strain energy reaches the maximum value of 668.2 J at a strain of 2.04% and then decreases rapidly as the strain increases. The peak values of the strain energy are 585.0, 535.9, and 395.72 J at RBPs of 40%, 55%, and 70%, respectively. At an RBP of 70%, as the strain increases, the strain energy does not decrease significantly after reaching its maximum value. The following can be easily observed from the pattern of strain energy. In a given S-RM specimen, the strain is caused primarily by the deformation of the soil, because the rock blocks undergo no deformation. At a low RBP, the deformation of an S-RM specimen is caused mainly by the deformation between the soil particles, so its strain energy increases rapidly as the axial strain increases. After reaching its maximum, its strain energy decreases rapidly as the axial strain increases, due to the debonding between the soil particles. At a high RBP, the rock blocks tend to support each other, resulting in a small amount of strain energy accumulating in the soil. However, a high RBP leads to a high rate of increase in the strain energy.

As shown in Figure 12, at the initial stage of the loading process, the rock blocks are not in contact with each other, so there is no friction between them and no strain energy. Although there is friction between soil and rock blocks, according to the previous studies [33], the friction between soil and rock blocks in the S-RM is much smaller than that between rock blocks, so it can often be ignored in the deformation of S-RM. As the strain increases, the rock blocks in each specimen gradually come in contact and mesh with each other, which is reflected by an increase in the frictional work. After the frictional work reaches a certain value, its rate of increase begins to level off, suggesting that the relative positions of the rock blocks are relatively fixed after the adjustment, and that the rock blocks only slide relative to each other. At a low RBP, it takes a long time for the rock blocks to come into contact with each other, and their frictional work is low.

## 4. Conclusions and Discussion

As a type of highly nonhomogeneous geological material, S-RMs display highly unique physical and mechanical properties and deformation and failure characteristics. In this study, using PFC^2D^ software, four S-RM specimen models with different RBPs (25%, 40%, 55%, and 70%) for biaxial testing were established, and their mechanical properties were investigated through a series of simulations. The following conclusions can be drawn:

1. The deformation and failure patterns observed in the S-RM specimens show that the strength of an S-RM increases as the RBP increases. At the residual stress stage, notable stress jumps occur due to the interaction between the rock blocks. The shear dilatancy of an S-RM increases with the RBP.
2. A high RBP leads to a wide, discontinuously distributed shear zone, a complex and dispersed crack distribution pattern, and contacts and collisions between the rock blocks surrounding the shear zone. This phenomenon occurs because the presence of the rock blocks prevents crack propagation and coalescence, resulting in a complex S-RM failure mode.
3. An analysis of the rotation of the rock blocks shows the following. At each RBP, rock blocks undergoing notable rotation are concentrated primarily near the shear zone. As the RBP increases, the shear zone expands. All the rock blocks outside the shear zone rotate by very small angles. The width of the shear zone can be determined from the rotation of the rock blocks. The number of rock blocks that undergo shear rotation under loading increases significantly as the RBP increases. The simulation results of this study show that most of the rock blocks rotate by small angles, mainly ranging from −5° to 5°.
4. Similar strain energy–axial strain relations were observed in the S-RM specimens with different RBPs. Specifically, the strain energy first increases and then decreases as the axial strain increases, similar to the stress–strain relation. In addition, the strain energy decreases as the RBP increases. At an RBP of 25%, the strain energy reaches the maximum value of 668.2 J at a strain of 2.04% and then decreases rapidly as the strain increases. The peak values of the strain energy are 585.0, 535.9, and 395.72 J at RBPs of 40%, 55%, and 70%, respectively. At an RBP of 70%, as the strain increases, the strain energy does not decrease significantly after reaching its maximum value. The lower the RBP, the longer it takes for the rock blocks to come into contact with each other, and the less the corresponding frictional work is.

The biaxial simulation test of earth rock mixture in this paper is relative to previous research results. The simulation results have been consistent with the relevant experiment conclusions [6,33], but they can more intuitively reflect the interaction and evolution mechanism of internal materials. In the follow-up work, we will conduct experiments and conduct detailed comparative research. At present, only the influence of the most important structural characteristic of different rock block proportions was considered. In order to simulate the deformation and failure mechanism of S-RM more accurately, further research can be considered from the aspects of rock block stone distribution, rock block direction, rock block particle size composition, soil and rock bond strength, etc. Due to the random nature of the investigated arrangements, sensitivity analysis is not considered in this paper, however, this is also worthy of further study.

## Figures and Tables

**Table 1:** Parameters used in numerical analyses.

Type	Unit Weight $\gamma \;\left(kN/m^{3}\right)$	Young’s Modulus *E* $\left(MPa\right)$	Poisson’s Ratio υ	Cohesion $c\;\left(kPa\right)$	Friction Angle $\varphi \;\left({}^{{}^{\circ}}\right)$	Tensile Strength $\sigma^{t}\;\left(kPa\right)$
Soil	21.6	50	0.3	49	16.9	25
Bedrock	27.0	19,000	0.20	820	43	809

**Table 2:** Meso-mechanical parameters of soil.

Parameters	Description	Value
ρ	Particle density ($kg/m^{3}$)	2160
$R_{max}$	Maximum particle radius (*m*)	0.6
$R_{max}/R_{min}$	Particle radius ratio, uniform distribution	2
$k_{n}$	Normal contact stiffness (*MPa*)	20
$k_{n}/k_{s}$	Normal-to-shear stiffness ratio	2
$F_{t}^{c}$	Tensile strength (*kN*)	3
$F_{s}^{c}$	Shear strength (*kN*)	3
$f_{s}$	Friction coefficient t (ball-ball/ball-rock)	0.12

## Data Availability

The data is available within the article and can be requested from the corresponding author.
